# Effects of Cigarette Smoke on Adipose and Skeletal Muscle Tissue: In Vivo and In Vitro Studies

**DOI:** 10.3390/cells11182893

**Published:** 2022-09-16

**Authors:** Lei Wang, Lieke E. J. van Iersel, Charlotte E. Pelgrim, Jingyi Lu, Ingrid van Ark, Thea Leusink-Muis, Harry R. Gosker, Ramon C. J. Langen, Annemie M. W. J. Schols, Josep M. Argilés, Ardy van Helvoort, Aletta D. Kraneveld, Johan Garssen, Paul A. J. Henricks, Gert Folkerts, Saskia Braber

**Affiliations:** 1 Division of Pharmacology, Utrecht Institute for Pharmaceutical Sciences, Faculty of Science, Utrecht University, 3584 CG Utrecht, The Netherlands or; 2 Department of Respiratory Medicine, NUTRIM School of Nutrition and Translational Research in Metabolism, Maastricht University Medical Centre +, 6200 MD Maastricht, The Netherlands; 3 Biochemistry and Molecular Biology of Cancer, Faculty of Biology, University of Barcelona, 08007 Barcelona, Spain; 4 Danone Nutricia Research, 3584 CT Utrecht, The Netherlands

**Keywords:** COPD, cigarette smoke, cachexia, adipose tissue, lipolysis, skeletal muscle

## Abstract

Chronic obstructive pulmonary disease (COPD), often caused by smoking, is a chronic lung disease with systemic manifestations including metabolic comorbidities. This study investigates adaptive and pathological alterations in adipose and skeletal muscle tissue following cigarette smoke exposure using in vivo and in vitro models. Mice were exposed to cigarette smoke or air for 72 days and the pre-adipose cell line 3T3-L1 was utilized as an in vitro model. Cigarette smoke exposure decreased body weight, and the proportional loss in fat mass was more pronounced than the lean mass loss. Cigarette smoke exposure reduced adipocyte size and increased adipocyte numbers. Adipose macrophage numbers and associated cytokine levels, including interleukin-1β, interleukine-6 and tumor necrosis factor-α were elevated in smoke-exposed mice. Muscle strength and protein synthesis signaling were decreased after smoke exposure; however, muscle mass was not changed. In vitro studies demonstrated that lipolysis and fatty acid oxidation were upregulated in cigarette smoke-exposed pre-adipocytes. In conclusion, cigarette smoke exposure induces a loss of whole-body fat mass and adipose atrophy, which is likely due to enhanced lipolysis.

## 1. Introduction

Chronic obstructive pulmonary disease (COPD) is a leading cause of death worldwide and smoking is by far its most important risk factor [1]. COPD is a chronic and complex systemic disease which can lead to manifestations beyond the lungs with systemic interactions over time [2]. Cachexia is a wasting syndrome commonly observed in a subgroup of COPD patients, which is characterized by progressive unintended weight loss (including both fat mass and skeletal muscle mass), causing further detrimental effects on disease progression and strongly associating with mortality [3,4]. The extent of emphysema in COPD patients is correlated with weight loss and fat loss [5]. Generally, the loss of fat mass observed during cachexia is not related to a loss of fat cells, but the decrease in cellular lipid content [6]. Enhanced lipid metabolism and triglyceride hydrolysis are the major metabolic pathways involved in the initiation and progression of cancer cachexia [7]. Lipolysis is a metabolic process through which triacylglycerol (TAGs) breaks down via hydrolysis into glycerol and free fatty acids (FFAs). These fatty acids are utilized throughout the body for heat and energy supply [8]. Lipases participating in lipolysis can play an essential role in the development of cancer-associated cachexia by breaking down the stored fat during lipolysis [7]. These processes may also be affected in patients with COPD. Adipose tissue is an important systemic organ in modulating a range of local and systemic metabolic and inflammatory pathways by the release of different bioactive factors, including cytokines, adipokines and hormones [9], which may contribute to respiratory disease progression [10]. Adipose tissue undergoes tissue remodeling, leading to enhanced lipolysis and the secretion of certain inflammatory cytokines, contributing to systemic inflammation [11]. Skeletal muscle wasting is one of the core symptoms of cachexia [12]; however, it is suggested that adipose tissue wasting probably occurs before the appearance of the reduction in lean mass [6,11]. The spill-over of systemic inflammation by the adipose tissue may be one of the underlying mechanisms involved in the depletion of skeletal muscle mass [13]. Reduced skeletal muscle mass is present in 4% up to 39% of patients with COPD [14], which is the consequence of an imbalance between processes of muscle protein synthesis and breakdown [15]. Moreover, it has been reported that mitochondrial quality control is dysregulated in cancer cachexia in tumor-bearing mice prior to skeletal muscle atrophy, showing an enhanced activity of the ubiquitin-proteasome pathway [16]. In patients with COPD it is well-established that resting metabolic rate may be elevated, contributing to a disturbed whole body energy balance that ultimately causes weight loss [17], but abnormalities in adipose tissue and the effects of cigarette smoke on lipolysis are relatively unexplored. Furthermore, it is unclear whether disturbances in adipose tissue metabolism are intertwined with altered muscle maintenance regulation or could be a putative driver of skeletal muscle catabolism. Therefore, in this manuscript, the effect of cigarette smoke exposure on adipose and muscle tissue in vivo using a murine model of COPD was investigated. In addition, 3T3-L1 preadipocytes were exposed to cigarette smoke total particulate matter (TPM) to further understand the direct effects of cigarette smoke exposure on lipid metabolism in vitro. 

## 2. Materials and Methods

### 2.1. In Vivo Study

#### 2.1.1. Animals

Specific-pathogen free female Balb/c mice, 11-13 weeks old (N = 15/16 per group) were obtained from Charles River Laboratories [18,19]. The mice were housed in groups (3 or 4 animals/cage) in filter-topped Makrolon cages (22 cm×16 cm×14 cm, floor area 350 cm^2^, Tecnilab-BMI, Someren, The Netherlands) with wood-chip bedding (Tecnilab-BMI, Someren, The Netherlands) and tissues (VWR, The Netherlands) available as cage enrichment. The mice were kept under standard conditions on a 12 h light/dark cycle (lights on from 7.00 am to 7.00 pm) at controlled relative humidity (relative humidity of 50–55%) and temperature (21 ± 2 °C) at the animal facility of Utrecht University. Food (AIN-93M, SNIFF) and water were available ad libitum. The in vivo study described in this article is part of a larger trial, including an air control group, smoke exposure group, and 6 other groups [20]. In accordance with the purpose of this study, investigating the effect of cigarette smoke exposure on both adipose and muscle tissue weight and function, the results of the analyses of the control and cigarette smoke exposure groups were investigated. All animal procedures described in this study were approved by the Ethics Committee of Animal Research of Utrecht University, Utrecht, The Netherlands (AVD1080020184785), and were conducted in accordance with the governmental guidelines. 

#### 2.1.2. Cigarette Smoke Exposure 

The mice were exposed in whole-body chambers to mainstream cigarette smoke or air for 72 consecutive days by peristaltic pump (SCIQ 232, Watson-Marlow 323, USA). Research cigarettes (3R4F) were obtained from the Tobacco Research Institute (University of Kentucky, Lexington, Kentucky) and filters were removed before use. The smoke chamber was connected to a peristaltic pump and vacuum to control the air circulation. The mice were exposed to cigarette smoke once a day, 7 days a week for 72 consecutive days, except on day 42, 52 and 62 (on these days, the mice received saline via intra-tracheal instillation, as they served as controls in a larger trial) using 4 cigarettes on day 1; 6 cigarettes on day 2; 8 cigarettes on day 3; 10 cigarettes on day 4; 12 cigarettes on day 5; and 14 cigarettes from day 6 to the end of the study. The speed of the peristaltic pump was kept at 35 rpm and the CO levels ranged between 200 and 400 ppm [19]. The mice were killed on day 73, as previously described [20]. 

#### 2.1.3. Body Composition 

After the last smoke exposure, the body weight was determined using a weighing scale and fat and lean mass were measured by using the EchoMRI Scan (Houston, TX, USA). 

#### 2.1.4. Grip Strength and Muscle Weight 

After the last smoke exposure, grip strength was measured as forelimb grip strength by a calibrated grip strength tester (Panlab, Cornella, Spain); the absolute average strength and absolute maximum strength were recorded over five repetitions [21]. Tibialis, soleus, extensor digitorum longus (EDL) and gastrocnemius muscle tissues were harvested; muscle tissues were weighed and snap-frozen for the following measurements.

#### 2.1.5. H&E Staining of Adipose Tissue and Determination of Fat Cell Number and Size 

The inguinal and para-ovary white adipose tissues were harvested, and the left side tissues were fixed in 10% formalin. Tissues were embedded in paraffin (Tissue processor, Leica); 5 μm sections were cut using a microtome (Leica RM 2165) and mounted on glass slides (StarFrost adhesive slides, Knittelgläser, Germany). The slides were deparaffinized and used for hematoxylin and eosin (H&E) staining according to the standard protocols. Microscopic images were taken using an Olympus BX50 microscope (Olympus, Tokyo, Japan). The number and size of fat cells were analyzed by ImageJ. 

#### 2.1.6. Immunofluorescent Staining Adipose Tissue

The tissues were placed on glass slides, deparaffinized and rehydrated in decreasing concentrations of ethanol and incubated with 0.3% H_2_O_2_/methanol for 30 min to quench endogenous peroxidase activity. Thereafter, the slides were incubated with rabbit-anti-CD 68 primary antibody (1:150; #97778S, Cell Signaling Technology, Leiden, The Netherlands) overnight at 4 °C after blocking with 5% goat serum in PBS containing 1% bovine serum albumin (BSA). After three-times washing steps with PBS containing 0.2% Tween 20 (PBST; pH 7.4), the slides were incubated with Alexa fluorescently conjugated goat anti-rabbit secondary antibody (1:200; Invitrogen, The Netherlands) for 2 h at room temperature. The nuclei were stained by Hoechst (1:2000; Invitrogen, USA) and the slides were rinsed after the Hoechst staining and mounted with FluorSave reagent (Merk Millipore, St. Louis, MO, USA). Rabbit IgG (1:150; #ab176094, Abcam, Cambridge, UK) was used as isotype control. Images were captured by the confocal microscope (TCS SP8 X, Leica, Germany).

#### 2.1.7. Adipose Tissue Homogenates for Cytokine Measurement

The inguinal and para-ovary white adipose tissues were harvested, and the right-side tissues were homogenized with cold 1% Triton X-100 (Sigma-Aldrich)/PBS solution containing protease inhibitors (Complete Mini, EDTA-free Protease Inhibitor Cocktail, Sigma-Aldrich, The Netherlands) by Precellys 24 tissue homogenizer (Bertin Technologies, France) at 4 °C. Thereafter, homogenates were centrifuged (15,000× *g*, 10 min, 4 °C) and the supernatant was collected. The protein concentration of the samples was measured using the Pierce BCA protein assay kit (Thermo Fisher Scientific, Waltham, MA, USA) according to the manufacturer's instructions. The samples were diluted to 2 mg of protein/mL and stored at −20 °C until further analyses. IL-6, TNF-α and IL-1β were measured by ELISA (Mouse IL-6 (#88-7064-88), TNF-α (#88-7324-88) and IL-1β (#88-7013-88) ELISA kit; Thermo Fisher Scientific, Waltham, MA, USA) according to the manufacturer’s instruction. 

#### 2.1.8. Leptin and KC Levels in Serum 

Blood was obtained by heart puncture and collected in Mini collect tubes (Greiner Bio-One, Alphen aan den Rijn, The Netherlands) on day 72 ± 18 h after the last air or smoke exposure. Blood samples were centrifuged (14,000× *g* for 10 min) and serum was stored at −20 °C for future use. Leptin and KC levels were determined by a quantitative Milliplex Luminex assay kit (ProcartaPlex, Thermo Fisher Scientific, Austria) according to the manufacturer’s instructions and using Luminex 200TM with Xponent software.

#### 2.1.9. RNA Isolation and Quantitative Real-Time PCR (qRT-PCR) for Skeletal Muscle Tissue 

##### RNA Preparation

Total RNA was isolated and extracted from muscle tissue using the RNeasy Mini Kit according to the manufacturer’s protocol (Qiagen, Germany). RNA integrity and quantitation were assessed using the RNA Nano 6000 Assay Kit of the Bioanalyzer 2100 system (Agilent Technologies, Palo Alto, CA, USA). 

##### qRT-PCR

cDNA was synthesized by the Tetro cDNA Synthesis Kit (Meridian, TN, USA) according to the manufacturer’s protocol in the T100 thermal cycler (Bio-Rad Laboratories, Hercules, CA, USA). A mixture of specific forward and reverse primers, SYBR® Green Supermixes for Real-Time PCR (Bio-Rad Laboratories or Meridian USA) and samples were prepared. Amplifications were performed according to the manufacturer’s instructions using LightCycler 480II (Roche, USA). Primers (Table S1) were commercially manufactured (Sigma-Aldrich, St. Louis, MO, USA). The mRNA quantity was calculated relative to the expression of the average value of GeNorm of Cyclo, RPLP0, HPRT, B2M, GUSB, YWHAZ. 

#### 2.1.10. Western Blot for Skeletal Muscle Tissue

Muscle tissue (soleus) from the mice was isolated. Proteins were isolated from the muscle tissue by homogenization in IP-buffer and subsequently heat denatured in Laemmli buffer (Sigma-Aldrich, Zwijndrecht, The Netherlands) for the separation by polyacrylamide gel electrophoresis, as previously described [22]. Blots were blocked with 5% milk powder in PBST (0.1% Tween 20 in PBS) at room temperature for 1 h and incubated with primary antibodies (p)-S6 Ribosomal protein, (p)-4EBP1, LC3B-I(II), 1:1000, Cell Signaling Technology, MA, USA) at 4 °C overnight, followed by washing blots in PBST. Goat Anti Rabbit IgG ((p)-S6, (p)-4EBP1, LC3B-I(I), 1:5000, Vector Laboratories, California, USA) were applied for 1h incubation at room temperature. Membranes were incubated with ECL Western blotting substrates (Bio-Rad Laboratories, Hercules, CA, USA) prior to obtaining the digital images. The digital images were acquired with the Molecular Imager Gel Doc XR system (Bio-Rad Laboratories, Hercules, CA, USA). Western blots were normalized by Ponceau-S staining. 

### 2.2. In Vitro Study

#### 2.2.1. T3-L1 Preadipocyte Cell Culture

The 3T3-L1 cell line (ATCC) was maintained in high glucose (4.5 g/L) Dulbecco’s modified Eagle’s medium (DMEM, Thermo Fisher Scientific, The Netherlands) supplemented with 10% (*v*/*v*) fetal bovine serum (FBS, Thermo Fisher Scientific, São Paulo, Brazil), 100 U/mL of penicillin (Sigma-Aldrich, The Netherlands) and 100 μg/mL of streptomycin (Sigma-Aldrich, The Netherlands) at 5% CO_2_ and 37 °C. Cells at passage 6—8 were used in this study. Cells were passaged at 80% confluence using a solution of 0.05% trypsin and 0.5 mM of EDTA. 

#### 2.2.2. TPM Preparation

TPM was collected by pumping the mainstream cigarette smoke of 3R4F research cigarettes through a peristaltic pump (SCIQ 232, Watson-Marlow 323, USA). TPM was collected by the type A/E glass fiber filter (PALL Life Sciences, Mexico), and the obtained TPM was dissolved in dimethyl sulfoxide (DMSO, Sigma-Aldrich, Zwijndrecht, The Netherlands) to a yield stock concentration of 50 mg/mL. This stock concentration was diluted to different concentrations, including 1.5625, 3.125, 6.25, 12.5, 25 and 50 µg/mL for the following experiment.

#### 2.2.3. Cell Viability and Cytotoxicity Assay

3T3-L1 pre-adipocytes were seeded and grown on 96-well plates until visual confluency was reached by microscopic inspection. Cells were exposed to different TPM concentrations for 24 h. To quantify the cell viability, after removing supernatants and rinsing the cells with warm PBS, 200 µL of the 0.5 mg/mL MTT labelling reagent was added into each well and incubated at 37 °C and 5% CO_2_ covered by aluminum foil for 2.5–3 h. A total of 100 µL of DMSO was added to each well after emptying the plate. When the crystals were solubilized entirely, the absorbance was measured using a microplate reader (Glomax discover, Promega) at 570 nm. To quantify the integrity of the cell membranes, the release of LDH into the growth media was measured after exposure to TPM. To this end, 50 µL of supernatant was incubated with a 50 µL well-mixed catalyst and dye substrate mixture (46:1) for 30 min at room temperature. The assay was terminated by adding 25 µL of stop solution, and the absorbance was measured using the microplate reader (Glomax discover, Promega) at 490 nm [23].

#### 2.2.4. Cell Differentiation and TPM Treatment

To induce the differentiation of preadipocytes to mature adipocytes, confluent 3T3-L1 pre-adipocytes cultured in phenol red-free DMEM (Thermo Fisher Scientific, The Netherlands) were treated with 0.5 mM of 3-sobutyl-1-methylxanthine (IBMX, Sigma-Aldrich, The Netherlands) that was dissolved in 1 mM of potassium hydroxide solution and 1 µg/mL of insulin (Sigma-Aldrich, The Netherlands) during day 0 until day 1, in order to induce cell differentiation (differentiation induction period). Maintenance medium (phenol-free DMEM supplemented with 1 µg/mL of insulin) was added and refreshed on day 2, 4 and day 7 (differentiation maintenance period) until day 8. During these 8 days, TPM, DMSO (for control group) or 100 mΜ of rosiglitazone (Sigma-Aldrich, The Netherlands) as a positive control was administrated to the cells simultaneously with the differentiation inducer (insulin or insulin with IBMX). TPM concentrations (6.25, 12.5 and 25 µg/mL) were selected based on the cell viability and cytotoxicity assays. TPM and rosiglitazone were dissolved in DMSO (0.1% *v*/*v*); therefore, this concentration of DMSO was added to the undifferentiated control (UDC) and differentiated control (DC) cells. After these 8 days of differentiation, supernatant or cells were collected for the Nile red staining, glycerol measurement, qPCR and Western blot analyses. See Figure S1 for the detailed overview of the cell culture procedures. 

#### 2.2.5. Nile Red Staining 

On day 8, after removing the supernatants, the cells were washed twice with PBS and fixed with 10% formalin at room temperature for 15 min. Deionized water was added for background measurement. Thereafter, the cells were incubated with the mixture of 0.1% Nile red (Sigma-Aldrich, The Netherlands), a fluorescent probe for the detection of the intracellular lipid content, and 0.05% Hoechst dye (Invitrogen, Carlsbad, CA, USA) for 20 min in the dark. The fluorescence intensity was measured by using a microplate reader (Glomax discover, Promega). The morphology images were captured by confocal microscope (Leica TCS SP8 X).

#### 2.2.6. Free Glycerol, FFAs and ATP Assays 

Cell culture supernatants (25× dilution) were used to test the levels of free glycerol release and FFAs release from cells using the free glycerol assay kit (#ab65337, Abcam, The Netherlands) and FFAs assay kit (#ab65341, Abcam, The Netherlands). Cells lysates were used for ATP measurement by the ATP assay kit (#MAK190-1KT, Sigma-Aldrich, The Netherlands). All the assays were performed according to the manufacturer’s instructions.

#### 2.2.7. RNA Isolation and Quantitative Real-Time PCR (qRT-PCR)/Gene Sequence

##### RNA Preparation

Total RNA was isolated and extracted from the cells using the RNeasy Mini Kit according to the manufacturer’s protocol (Qiagen, Germany). RNA integrity and quantitation were assessed using the RNA Nano 6000 Assay Kit of the Bioanalyzer 2100 system (Agilent Technologies, Palo Alto, CA, USA). RNA degradation and contamination were monitored on 1% agarose gels (Figure S2). 

##### qRT-PCR

cDNA was synthesized by an iScript TM advanced kit (Bio-Rad, Hercules, CA, USA) (adipocytes) according to the manufacturer’s protocol in the T100 thermal cycler (Bio-Rad Laboratories, Hercules, CA, USA). A mixture of specific forward and reverse primers, SYBR® Green Supermixes for Real-Time PCR (Bio-Rad Laboratories or Meridian USA) and samples were prepared, and amplifications were performed according to the manufacturer’s instructions using the CFX96 Touch™ Real-Time PCR Detection System (Bio-Rad Laboratories, Hercules, CA, USA). Primers (Table S1) were commercially manufactured (Biolegio BV, Nijmegen, Netherlands). The specificity and efficiency of the primers were tested by a temperature gradient, the melting curve was evaluated, and the optimum annealing temperature was determined. The mRNA quantity was calculated relative to the expression of β-actin.

##### mRNA Non-Directional (polyA)

RNA samples were used for library preparation using NEB Next® Ultra RNA Library Prep Kit for Illumina ®. Indices were included to multiplex multiple samples. Briefly, mRNA was purified from total RNA using poly-T oligo-attached magnetic beads. After fragmentation, the first strand cDNA was synthesized using random hexamer primers, followed by the second strand cDNA synthesis. The library was ready after end repair, A-tailing, adapter ligation and size selection. After amplification and purification, the insert size of the library was validated on an Agilent 2100 and quantified using a q-PCR. Libraries were then sequenced on Illumina NovaSeq 6000 S4 FlowCell with PE150 according to results from library quality control and expected data volume. Library preparation, sequencing and analysis was performed by Novogene (Cambridge, UK) Company Limited.

#### 2.2.8. Western Blot

After the differentiation procedure, 3T3-L1 cell lysates were collected by adding RIPA cell lysis buffer (Thermo Fisher Scientific, Waltham, MA, USA) containing protease inhibitors (Roche Applied Science, Pennsburg, Germany). Total protein concentrations were determined using a Pierce BCA protein assay kit (Thermo Fisher Scientific, Waltham, MA, USA) according to the manufacturer’s instructions. A total of 30 μg of protein sample was loaded onto polyacrylamide gels (4–20% Tris–HCl, Bio-Rad Laboratories, Hercules, CA, USA) that were separated using electrophoresis, and electro transferred onto polyvinylidene difluoride membrane (Bio-Rad Laboratories, Hercules, CA, USA) using the Trans-Blot Turbo system (Bio-Rad Laboratories, Hercules, CA, USA). Blots were blocked with 5% milk powder in PBST (0.1% Tween 20 in PBS) at room temperature for 1 h and incubated with primary antibodies (p-)HSL, HSL, β-actin, (1:1000, Cell Signaling Technology, MA, USA) at 4 °C overnight, followed by washing the blots in PBST. Corresponding horseradish peroxidase-coupled secondary antibodies from Dako (for p-HSL, HSL, β-actin) (1:10,000, Agilent Technologies, Santa Clara, CA, USA) were applied for 1h incubation at room temperature. Membranes were incubated with ECL Western blotting substrates (Bio-Rad Laboratories, Hercules, CA, USA) prior to obtaining the digital images. The digital images were acquired with the Molecular Imager Gel Doc XR system (Bio-Rad Laboratories, Hercules, CA, USA).

### 2.3. Statistical Analysis

The results are presented as mean ± SEM. The student *t*-test and spearman tests (analyses of correlation) were conducted for in vivo results. The in vitro results from different groups were statistically determined by a one-way ANOVA, followed by Tukey’s multiple comparison post hoc test. The difference was considered statistically significant at *p* < 0.05. All statistical analyses were conducted using GraphPad Prism (version 8.3). The required sample size was calculated with G*Power v 3.1.9 based on a power of 80% and α = 0.05 and a primary outcome parameter derived from previous observations.

## 3. Results

### 3.1. COPD-Related Characteristics, Body Composition, Food Intake, Leptin and Cytokine Levels in Serum 

The mean linear intercept (Lm), a measure of interalveolar wall distance representing lung tissue damage, and the BALF cell numbers (macrophages, neutrophils and lymphocytes) and cytokines were significantly increased after cigarette smoke exposure as compared to the air-exposed group [20,24]. These findings confirm that cigarette smoke exposure induced COPD-related characteristics. 

**Figure 1:**
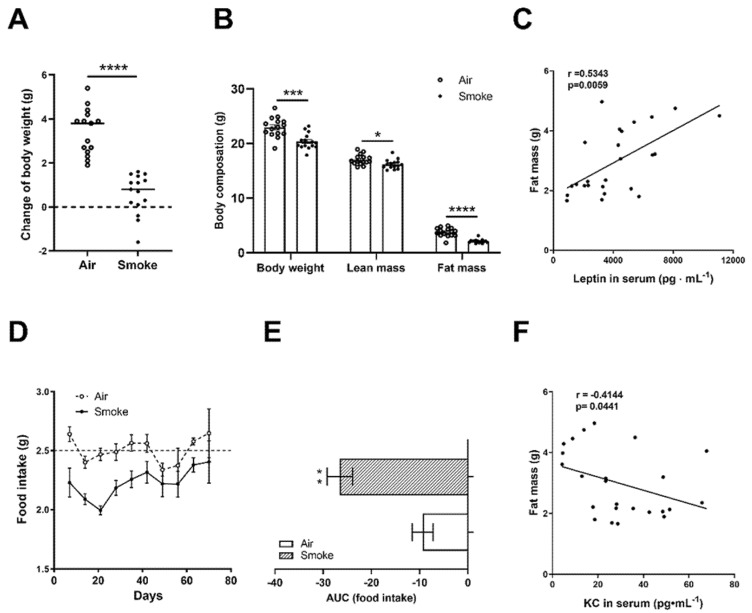
Body composition, food intake, leptin and cytokine levels in serum. Body weight changes (from day 1 to day 72) (**A**) and body composition at the endpoint (body weight, lean mass and fat mass) (**B**) were quantified. Correlations between fat mass and leptin levels in serum (**C**) were analyzed using Spearman correlation. Food intake was recorded every week (**D**) and the area under the curve of food intake (**E**) was analyzed. Correlation between fat mass and KC levels (**F**) were analyzed using Spearman’s correlation. N = 12–16 mice/group. Values are represented as mean ± SEM. * *p* < 0.05, ** *p* < 0.01, *** *p* < 0.001, **** *p* < 0.0001; smoke group compared with air group.

The average body weight between the groups did not differ before starting the exposures. The body weight gain (from day 1 to day 72) in the cigarette smoke-exposed mice was significantly lower compared to the air-exposed animals (Figure 1A). At the endpoint (day 72), the echo MRI showed a lower body weight in cigarette smoke-exposed mice (Figure 1B). The difference in body weight between the groups could be explained by a proportional loss in lean mass of 4.9%, as well as fat mass of 43.8% (Figure 1B). Cigarette smoke exposure significantly decreased serum leptin levels [24], and the leptin levels were positively correlated with fat mass (Figure 1C). Food intake data were collected from day 7 and recorded weekly; smoke exposure clearly affected the appetite of the mice, as demonstrated by a lower food intake (Figure 1D). The area under the curve (AUC) for food intake in the cigarette smoke-exposed mice was decreased as compared to air-exposed mice (Figure 1E). KC levels were measured in the serum, which were increased after exposure to the cigarette smoke [20], and KC levels were negatively correlated with the fat mass (Figure 1F).

### 3.2. Histomorphological Changes in Adipose Tissue

**Figure 2:**
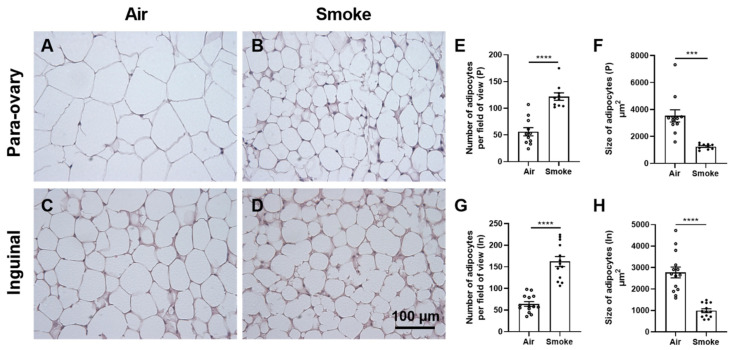
Histomorphological changes in adipose tissue. Para-ovary (**A**,**B**) and inguinal (**C**,**D**) white adipose tissues were collected and stained with H&E (scale bar = 100 μm). The number (**E**) and size (**F**) of adipocytes of para-ovary white adipose tissue, and the number (**G**) and size (**H**) of adipocytes of inguinal white adipose tissue were determined by ImageJ. Values are expressed as mean ± SEM. *** *p* < 0.001, **** *p* < 0.0001, smoke group compared with air group; N = 10—14 mice/group.

To investigate the morphological changes in adipose tissue, two different types of white adipose tissue (para-ovary and inguinal adipose tissue) were isolated and stained with H&E. Representative histological pictures of para-ovary adipose (Figure 2A,B) and inguinal adipose tissue (Figure 2C,D) of air- (Figure 2A,C) and cigarette smoke-exposed mice (Figure 2B,D) are depicted. Cigarette smoke exposure significantly increased the number (Figure 2E,G) and decreased the size of adipocytes (Figure 2F,H) in both para-ovary and inguinal adipose tissues. 

### 3.3. Macrophages and Pro-Inflammatory Cytokines in Adipose Tissue

**Figure 3:**
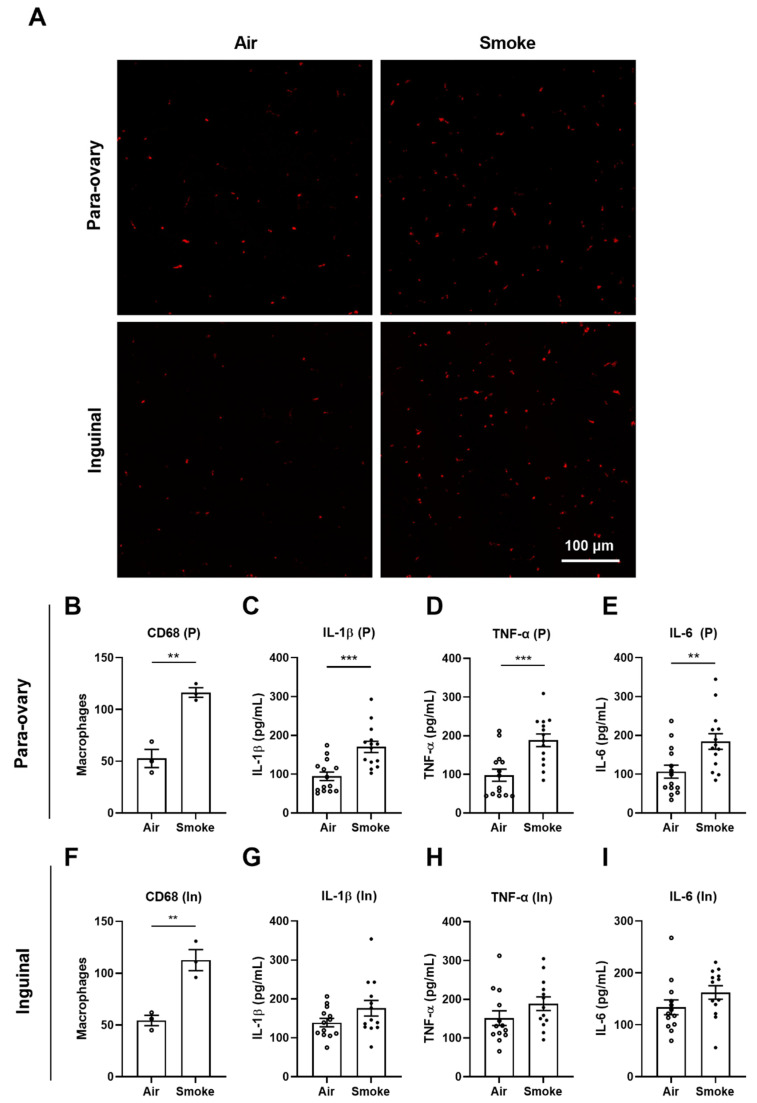
Macrophages and pro-inflammatory cytokines in adipose tissue. The presence of macrophages in para-ovary and inguinal adipose tissues was investigated by immunofluorescence microscopy using anti-CD68 antibodies (**A**) (scale bar = 100 μm). The macrophages numbers of CD68 staining for para-ovary (**B**) and inguinal (**F**) adipose tissue were counted. N = 3 for each group. The levels of IL-1β (**C**), TNF-α (**D**) and IL-6 (**E**) in para-ovary adipose tissue homogenates and the levels of IL-1β (**G**), TNF-α (**H**) and IL-6 (**I**) in inguinal adipose tissue homogenates were determined by ELISA. Values are expressed as mean ± SEM. ** *p* < 0.01, *** *p* < 0.001, smoke group compared with air group; N = 13–14 mice/group were used for ELISA.

To investigate the inflammatory response induced by cigarette smoke exposure in adipose tissue, macrophage infiltration (staining with anti-CD68 antibodies) and pro-inflammatory cytokine (IL-1β,TNF-α and IL-6) concentrations were measured in both para-ovary and inguinal white adipose tissues. Representative pictures of CD68-positive macrophages are depicted in Figure 3A, while pictures of the isotype control and negative control are depicted in Figure S4. The number of CD68-positive cells is significantly increased in cigarette smoke-exposed mice (Figure 3B,F). Cigarette smoke exposure significantly increased the level of IL-1β, TNF-α and IL-6 (Figure 3C–E) in para-ovary adipose tissue. No significant differences in IL-1β, TNF-α and IL-6 levels were observed in inguinal adipose tissue (Figure 3G–I). 

### 3.4. Muscle Function, Muscle Weight, Mitochondrial and Protein Turnover Markers in Soleus Muscle 

**Figure 4:**
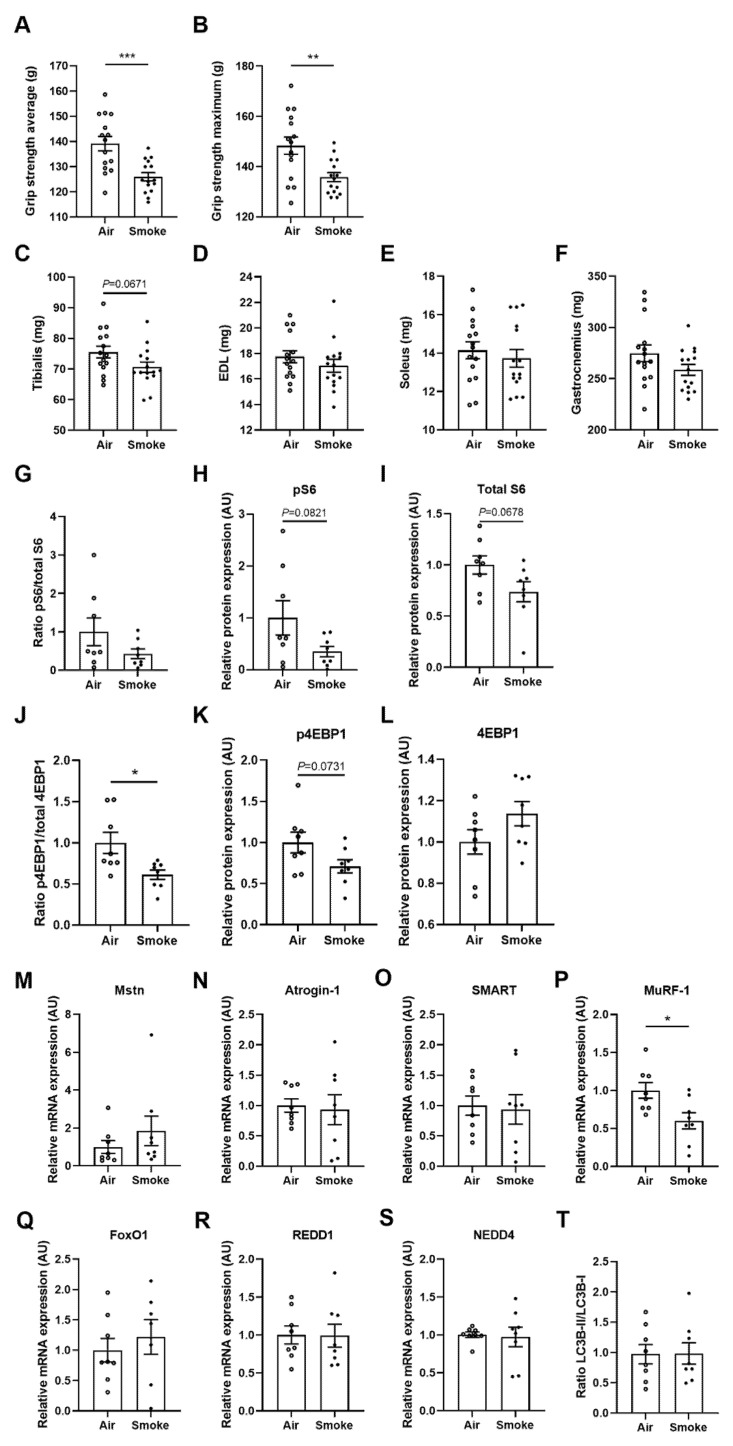
Muscle function, muscle weight, mitochondrial and protein turnover markers in soleus muscle. Grip strength was determined at day 72 and represented as the average values (**A**) and maximum values (**B**) of five measurements per mouse. Paired muscle weights of tibialis (**C**), EDL (**D**), soleus (**E**) and gastrocnemius (**F**) were determined. N = 15–16 mice/group. Protein expression of ratio pS6/total S6 (**G**), pS6 (**H**) S6 (**I**), ratio p4EBP1/total 4EBP1 (**J**), p4EBP1 (**K**), 4EBP1 (**L**) and ratio LC3BII/LC3BI was analyzed by Western blot (**T**). Gene expression levels of Mstn (**M**), Atrogin-1 (**N**), SMART (**O**), MuRF-1 (**P**), FoxO1 (**Q**) REDD1 (**R**) and NEDD4 (**S**) were assessed, normalized to GeNorm and expressed as fold change compared to control, N = 8 mice/group. Values are represented as mean ± SEM. * *p* < 0.05, ** *p* < 0.01, *** *p* < 0.001; smoke group compared with air group.

Grip strength was measured after the last time of cigarette smoke exposure to determine the muscle function. Cigarette smoke exposure significantly decreased the average and maximum grip strength (Figure 4A,B). Smoke exposure tends to decrease the weight of the tibialis (*p* = 0.0617, Figure 4C). No significant differences were observed in the weight of EDL, soleus and gastrocnemius (Figure 4D–F). The effect of cigarette smoke exposure on protein turnover signaling in the soleus muscle was investigated, including the protein levels of total ribosomal protein S6 (S6), phosphorylated S6 (pS6), total initiation factor 4E binding protein 1 (4EBP1) and p4EBP1. 4EBP1 phosphorylation (Figure 4J–L) was significantly reduced in the cigarette smoke-exposed group. In addition, the trend of reduced S6 phosphorylation (Figure 4G–I) was also observed in the smoke-exposed group. This indicated an attenuated protein synthesis signaling in the muscle of cigarette smoke-exposed mice. Furthermore, smoke exposure did not result in consistent and significant alterations in the mRNA levels of molecular markers involved in protein degradation, including the mRNA levels of myostatin (Mstn), Atrogin-1, f-box protein 21 (SMART), forkhead box O1 (FoxO1), DNA-damage-inducible transcript 4 (REDD1), and Neural precursor cell expressed, developmentally down-regulated 4 (NEDD4) (Figure 4M–O, Q–S) and protein levels of the ratio LC3BII/LC3BI (Figure 4T). Only the mRNA levels of Muscle Ring Finger protein 1 (MuRF-1) showed a significant decrease after smoke exposure (Figure 4P). Original blots are depicted in Figure S3. Furthermore, to investigate whether loss of muscle oxidative capacity was evident as it has been suggested to precede skeletal muscle atrophy, the molecular markers of mitochondrial biogenesis and content were measured in the soleus. Nuclear respiratory factor 1 (Nrf1) decreased significantly after smoke exposure compared to air control, and key regulator of biogenesis peroxisome proliferator-activated receptor-gamma coactivator 1-alpha (PGC-1α) tended to decrease after smoke exposure (*p* = 0.056). Further, no significant alterations were observed for mitochondrial biogenesis and oxidative phosphorylation complexes (Figure S5).

### 3.5. Lipid Accumulation and Leptin Levels in 3T3-L1 Pre-Adipocytes 

Based on the obvious fat mass decrease after cigarette smoke exposure in mice, 3T3-L1 pre-adipocytes were exposed to cigarette smoke TPM to further understand the effect of cigarette smoke exposure on lipid metabolism. Concentrations of TPM were determined by cell viability assay and used in the following experiment (Figure S6). 

**Figure 5:**
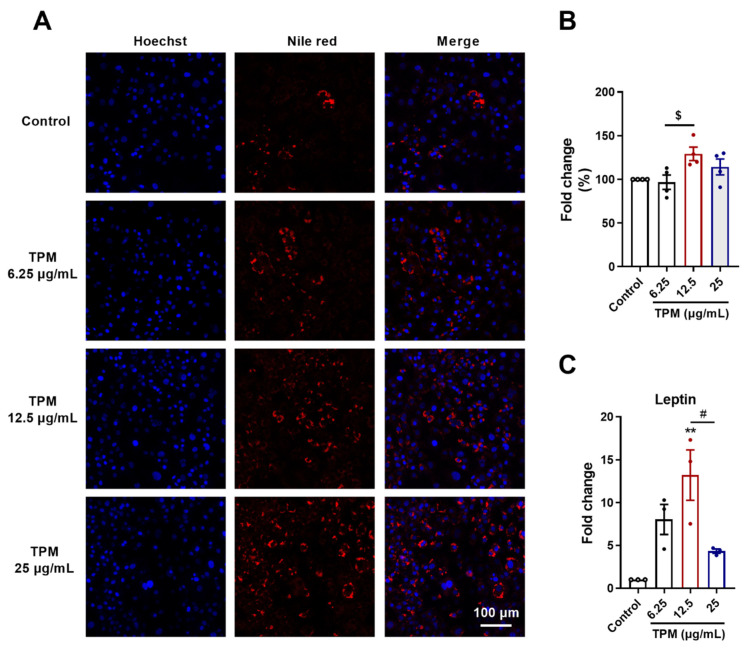
Lipid accumulation and leptin levels in 3T3-L1 pre-adipocytes. Different TPM concentrations (6.25, 12.5 and 25 μg/mL) were added to the 3T3-L1 pre-adipocytes during the differentiation process of 8 days. Nuclei and lipid droplets were stained with Hoechst and Nile red dye, respectively (**A**). Nile red fluorescent intensity was read and quantified (**B**), N = 4 replicates. mRNA expression levels of leptin were evaluated (**C**), N = 3 replicates. Values are expressed as mean ± SEM. ** *p* < 0.01, compared with control group; $ *p* < 0.05, compared with 6.25 μg/mL of TPM group; # *p* < 0.05, compared with 12.5 μg/mL of TPM group.

The different TPM concentrations were administered to the 3T3-L1 pre-adipocytes during the differentiation process (day 0 until day 8) (see also Figures S1 and S7 for more details). The cells were stained with a mixture of Nile red (intracellular lipid droplets) and Hoechst (nuclei) at day 8. Representative images are depicted in Figure 5A, and the fluorescent intensity of the red dye was quantified (Figure 5B). The percentage of lipid droplets was increased after the addition of 12.5 μg/mL of TPM compared to 6.25 μg/mL of TPM, but not to the control. The higher concentration of 25 μg/mL of TPM did not induce a significant effect on the percentage of lipid droplets compared to the control (Figure 5B). Leptin mRNA expression levels were analyzed. Although 6.25 μg/mL of TPM did not significantly affect the leptin mRNA levels, TPM (12.5 μg/mL) significantly increased the mRNA expression of leptin compared to the control cells, and this increase was significantly decreased by increasing the TMP concentration to 25 μg/mL (Figure 5C). 

### 3.6. Lipolysis and Fatty Acid Oxidation in 3T3-L1 Adipocyte

**Figure 6:**
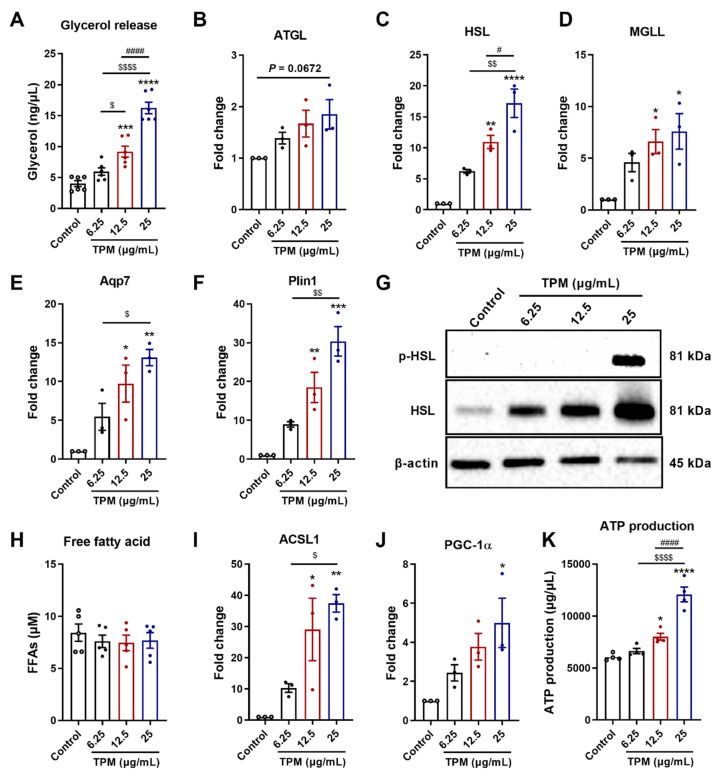
Lipolysis and fatty acid oxidation in 3T3-L1 adipocytes. Different TPM concentrations (6.25, 12.5 and 25 μg/mL) were added to the 3T3-L1 pre-adipocytes during the differentiation process of 8 days. Glycerol release (**A**) was determined in the supernatants (N = 6 replicates) and mRNA expression of ATGL (**B**), HSL (**C**), MGLL (**D**), Aqp7 (**E**), Plin1 (**F**), ACSL1 (**I**) and PGC-1α (**J**) were determined by qRT-PCR; the data were normalized to β-actin and were calculated relative to control (N = 3 replicates). FFAs (**H**) were determined in the supernatants (N = 5 replicates) and ATP production (**K**) was measured in the cell lysates (N = 4 replicates). Protein expression levels of HSL, phosphorylated HSL (phosphor-HSL) and β-actin (**G**) were analyzed by Western blot. Values are expressed as mean ± SEM, * *p* < 0.05, ** *p* < 0.01, *** *p* < 0.001 and **** *p* < 0.0001 compared with control group; $ *p* < 0.05, $$ *p* < 0.01 and $$$$ *p* < 0.0001 compared with 6.25 μg/mL of TPM group; # *p* < 0.05 and #### *p* < 0.0001 compared with 12.5 μg/mL of TPM group.

Lipolysis is an important step in lipid metabolism that regulates lipid mobilization. Glycerol as the final lipolysis product was determined. The concentrations of 12.5 and 25 μg/mL of TPM significantly increased the glycerol production compared to the control cells (Figure 6A). The increased glycerol release was supported by the mRNA expression of aquaporin 7 (Aqp7), the water-selective membrane channel responsible for glycerol efflux, which was also significantly increased by 12.5 and 25 μg/mL of TPM as compared to the control cells (Figure 6E). Furthermore, the mRNA expression of the enzymes involved in the lipolysis was increased by TPM exposure: 25 μg/mL of TPM tended to increase the mRNA expression of adipose triglyceride lipase (ATGL) (*p* = 0.0672, Figure 6B), and 12.5 and 25 μg/mL of TPM significantly upregulated the mRNA expression of hormone-sensitive lipase (HSL) (Figure 6C) and monoglyceride lipase (MGLL) compared to the control cells (Figure 6D). The mRNA expression level of perilipin 1 (Plin 1), a lipid droplet-associated protein that is required for the translocation of HSL from the cytosol to lipid droplets upon stimulation [25], was significantly upregulated by 12.5 and 25 μg/mL of TPM compared to the control cells (Figure 6F). Since HSL, a hormone-sensitive lipase, is crucial for the rate-limiting step in lipolysis, the protein level of HSL and the phosphorylation of HSL were also determined (Figure 6G and Figure S8). TPM at 12.5 and 25 μg/mL significantly increased the HSL protein expression, and 25 μg/mL of TPM increased phosphorylated HSL compared to the control cells. 

To further understand the effects of TPM on lipid metabolism, FFAs, another product liberated from lipolysis, were measured in the supernatant. Interestingly, no significant changes were found in FFA release after exposure to increasing concentrations of TPM (Figure 6H). The utilization of FFAs in mitochondria (fatty acid oxidation) was also determined. Acyl-CoA synthetase long chain family member 1 (ACSL1) is known to convert free long-chain fatty acids into fatty acyl-CoA esters, and thereby play a key role in lipid biosynthesis, as well as in fatty acid transport and degradation [26]. The mRNA expression levels of ACSL1 were elevated in 12.5 µg/mL and 25 µg/mL of TPM-exposed groups (Figure 6I). PGC-1α is associated with mitochondrial oxidative capacity, and PGC-1α mRNA levels were elevated in the 25 µg/mL of TPM group (Figure 6J). ATP production as the result of FFAs utilization for energy production was measured in the cells. TPM concentration dependently increased the ATP production, and 25 µg/mL of TPM had the most obvious effect on ATP production (Figure 6K). To further support these results, RNA-sequencing was performed, and the data showed that genes involved in mitochondrial oxidative phosphorylation were highly enriched after exposure to cigarette smoke. Mitochondrial respiratory complexes I, II, III, and IV were highly expressed in the mitochondrial oxidative phosphorylation pathway (Fold change >2, Figure S10). 

## 4. Discussion

About 25% of patients with COPD will develop cachexia [17], which is associated with excessive mortality [26]. Cachexia is a syndrome characterized by an involuntary loss of body weight. Although muscle wasting is the main clinical manifestation observed in cachexia, recent studies show that fat loss occurs more rapidly and more precociously than the decrease in lean mass in cancer cachexia [11]. 

Information about the effect of cigarette smoke exposure on adipose tissue, as well as the important role of adipose tissue in the interrelationship with muscle maintenance is scarce. Therefore, in this study, a murine cigarette smoke-induced COPD model was used to investigate the effects of cigarette smoke exposure on body composition (body weight, fat mass, lean mass), adipose tissue morphology and inflammation, as well as on muscle function and muscle mitochondrial and protein turnover markers. In addition, 3T3-L1 pre-adipocytes were exposed to TPM from cigarette smoke to further understand the effect of cigarette smoke exposure on lipid metabolism (lipolysis). 

In the in vivo study, COPD-like characteristics, including enlarged alveolar diameters and increased inflammatory cells in the lung [24], were observed, as well as a decreased body weight gain, which was detected after smoke exposure. It is recognized that weight and fat mass loss is common in many COPD patients, which is correlated with the extent of emphysema [5,27], whereas COPD patients with a more chronic bronchitis phenotype are more likely to gain weight [28]. In this present study, a cigarette smoke-induced decrease in lean mass and fat mass was found. The drop in fat mass was relatively more obvious than the drop in lean mass. A decrease in food intake was also found in the cigarette smoke-exposed group. A possible explanation might be that nicotine is a strong signal that suppresses appetite and decreases food intake, leading to reduced body weight [29].

Adipose (fat) tissue is the main source for producing leptin, which is involved in energy homeostasis and controls body weight via the regulation of appetite and energy expenditure [30,31]. Decreased serum leptin levels and decreased body weight are commonly found in many COPD patients [32]. Circulating leptin levels were found independent of the TNF-α system, and they were regulated physiologically even in the presence of cachexia in patients with COPD [33]. In this study, KC levels were negatively correlated with fat mass, indicating a link between systemic inflammation and fat mass loss. Leptin exerts multiple effects on the respiratory system, as this cytokine-like hormone plays important physiological roles. Leptin has the ability to modulate inflammation, which may integrate systemic inflammation [34]; therefore, the leptin-signaling pathway might be involved in this low systemic inflammatory response [35]. Nevertheless, the low inflammatory systemic response following cigarette smoke exposure may also be related to the “spill over” of the initial, localized pulmonary inflammatory response [20,36], as in the current murine model, exposure to cigarette smoke significantly enhanced the levels of CRP and KC in BALF as well as in serum, and a significant positive correlation between the level of KC in BALF and the amount of KC in serum was observed [20]. 

Adipose tissue wasting also occurred in cancer cachexia patients [11]. However, information about the changes in adipose tissue in cigarette smoke-induced COPD is scarce. Adipose tissue is currently viewed as a highly dynamic endocrine organ that is involved in a wide range of metabolic and inflammatory processes [37]. Visceral (para-ovary) adipose tissue is located around the vital organs and is commonly linked to metabolic disorders, while subcutaneous (inguinal) adipose tissue is located beneath the skin and is associated with beneficial metabolic effects [38]. In this in vivo study, the morphology of both para-ovary and inguinal adipose tissues showed a significant decrease in the size of adipocytes and increased the number of adipocytes (atrophy) after 10 weeks of cigarette smoke exposure. Within adipose tissue, adipocytes and macrophages are the major cell types that synthesize and release a large number of different proteins involved in inflammation, immunity, lipid metabolism and homeostasis, such as inflammatory cytokines and adipokines [37]. The number of macrophages were increased in both para-ovary and inguinal white adipose tissue in cigarette smoke-exposed mice. Inflammatory cytokines produced by adipose tissue contribute to adipose depletion [39], and we observed that IL-1β, IL-6 and TNF-α levels were increased in para-ovary and inguinal adipose tissue after cigarette smoke exposure. Interestingly, the increase in IL-1β, IL-6 and TNF-α levels induced by cigarette smoke exposure was more pronounced in para-ovary adipose tissue, which might be due to a difference in blood supply or susceptibility between these two different types of adipose tissue, as para-ovary adipose tissue is a perigonadal depot and inguinal tissue is a subcutaneous depot [38].

Until now, it has remained unclear whether abnormalities in adipose tissue and thereby enhanced systemic inflammation are a putative driver of skeletal muscle impairment. In this present murine model, cigarette smoke exposure decreased the maximum and average grip strength, indicating that muscle weakness was present. Skeletal muscle dysfunction is common in many COPD patients, which represents an important comorbidity that is associated with a poor health-related quality of life and reduced survival [40]. In addition, handgrip strength is associated with functional limitation and COPD-specific symptoms, as well as with higher exacerbation frequencies [41]. The observed muscle weakness could be related to the systemic release of the pro-inflammatory cytokines and adipokines produced in the adipose tissue [42], as higher levels of systemic inflammatory mediators are associated with lower muscle strength and skeletal muscle mass over time [13]. However, it cannot be excluded that systemic factors produced by other organs may also play a role in this process. 

A previous study showed that cigarette smoke exposure enhances proteolysis and inhibits protein synthesis, resulting in a depletion of muscle mass [43]. Although muscle weights were not significantly reduced in the current study, protein synthesis signaling was affected in the muscle of cigarette smoke-exposed mice, whereas other markers of protein turnover were similar in both cigarette smoke- and air-exposed mice. In the cigarette smoke-exposed animals in the current study, muscle contractile force (fore-limbs grip strength) was reduced, in support of developing a loss of muscle mass. Other than a loss of muscle mass, a loss of muscle oxidative phenotype or mitochondrial dysregulations can also be important drivers of muscle weakness. In a cancer cachexia mouse model, it has been shown that mitochondrial degeneration precedes cachectic muscle wasting in tumor-bearing mice [16]. Furthermore, 16 weeks of cigarette smoke exposure in mice increased the expression of the inflammatory cytokine TNF-α, and thereby suppressed the mRNA expression of PGC-1α, which may affect muscle function [44]. In patients with cancer-associated cachexia, dysfunctional mitochondria are associated with lower muscle strength and muscle atrophy [45]. In the current study, no obvious changes in the expression of molecular markers involved in the mitochondrial biogenesis and content were observed (except Nrf1) in the muscles of cigarette smoke-exposed mice when compared to control mice. It indicates that there might be an initial (premature) decrease in oxidative phenotype regulation in the muscle, based on the decrease in Nrf1 expression and the decreased PGC-1α trend, but there were no alterations in the downstream gene expression levels of TFAM and the oxidative phosphorylation complexes. The molecular signaling alterations in the muscle may depend on the duration of cigarette smoke exposure. In this study, 10 weeks of cigarette smoke exposure clearly affected adipose tissue, but the duration might not have been long enough to induce solid changes in the molecular markers of protein turnover or oxidative phenotype in skeletal muscle tissue, as seen after longer cigarette smoke exposure times [46,47,48]. In future experiments, an extended period of smoke exposure may, therefore, adequately elicit chronic effects of cigarette smoke exposure on skeletal muscle that clarify whether the systemic release of the pro-inflammatory cytokines and adipokines produced by the adipose tissue affect skeletal muscle.

In order to explore the potential mechanism for the fat mass loss and adipose atrophy in the cigarette smoke-induced COPD model, an in vitro model was used in which 3T3-L1 pre-adipocytes were exposed to TPM (from cigarettes smoke). Based on the Nile red staining, 12.5 μg/mL of TPM showed increased lipid droplets when compared to the 6.25 μg/mL of TPM. However, when the concentration of TPM reached 25 μg/mL, the lipid amount in the adipocytes decreased. Leptin levels corresponded with the amount of lipid droplets in the adipocytes. TPM affected lipid metabolism; however, contrasting results were obtained between 12.5 μg/mL and 25 μg/mL of TPM exposure, which might be related to changes in the balance between lipolysis (lipid consumption) and lipogenesis (lipid accumulation). It might be suggested that the lipolysis was enhanced after adding 25 μg/mL of TPM.

Changes in adipocytes size might be mainly dependent on cellular triglyceride content [49], and the loss of adipose tissue in cancer cachexia is partly the result of increased lipolysis [50]. In addition, increased lipolysis and fat oxidation, as well as impaired lipid deposition and adipogenesis, may underlie adipose atrophy in cancer cachexia [39]. All these findings indicate that lipolysis and fatty acid oxidation might play a pivotal role in fat loss and adipose atrophy. In this current study, glycerol, the end-product of lipolysis, was concentration dependently increased by TPM, which is indicative for an increased lipolysis. Adipose ATGL, HSL and MGLL are three major enzymes acting in sequence in the hydrolysis of TAG producing FFAs and glycerol. ATGL catalyzes the initial step of TAG hydrolysis, generating diacylglycerol (DAG) and one fatty acid. HSL is rate-limiting for the second step of TAG lipolysis converting DAG to one fatty acid and monoacylglycerol (MAG); this process involves the protein kinase A-dependent phosphorylation of HSL. MGLL is the key enzyme in the hydrolysis of the endocannabinoid 2-arachidonoylglycerol and converts MAG to a free fatty acid and glycerol. Plin 1 is known as a lipid droplet-associated protein and is also associated with the lipolysis process [51]. The mRNA expression levels of ATGL, HSL, MGLL and Plin 1 were all increased after TPM exposure in 3T3-L1 pre-adipocytes, and 25 μg/mL of TPM caused the most pronounced effect on lipolysis. In addition, HSL protein levels were also enhanced after TPM exposure, and 25 μg/mL of TPM induced an increase in the expression of phosphorylated HSL. Aqp7, a water selective membrane channel functioning as a glycerol transporter, was also highly enhanced by 25 μg/mL of TPM. Taken together, the results indicate that TPM in a concentration-dependent way promoted the lipolysis in 3T3-L1 pre-adipocytes. 

**Figure 7:**
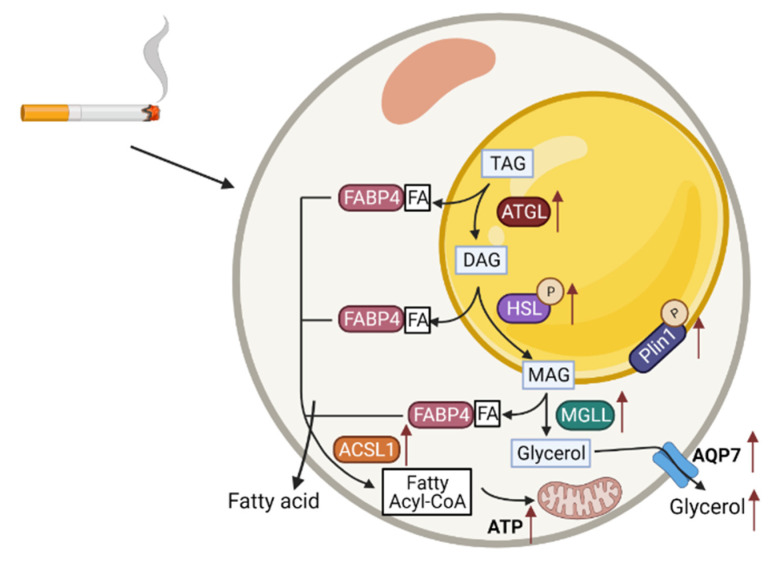
Molecular mechanisms of lipolysis in adipocytes and the effects of TPM exposure (red arrow). Lipolysis in adipocytes, the hydrolysis of triacylglycerol (TAG) to release fatty acids (FAs) and glycerol for use by other organs as energy substrates is a unique function of white adipose tissue. The activation of lipases including ATGL, HSL and MGLL is crucial for the initiation of lipolysis, and Plin1 phosphorylation is a key event in the activation of TAG hydrolysis. HSL phosphorylation promotes the translocation of the enzyme from the cytosol to the surface of the lipid droplet. Docking of adipocyte FA-binding protein 4 (FABP4) to HSL facilitates outflow from the cell of non-esterified fatty acid (NEFAs) released by the hydrolysis of TAGs. AQP7 is the water-selective membrane channel responsible for glycerol efflux. Released FFAs requires the activation via ACSL1 that converts FFAs to Fatty acyl-CoA. Converted Fatty acyl-CoA can be transferred to mitochondrial matrix and oxidized in mitochondria to produce ATP as the central part of the human energy metabolism. The lipolysis was enhanced after TPM exposure, as observed by increased lipases, released glycerol, as well as augmented fatty acid oxidation by increased ATP production.

The majority of studies have indicated elevated lipolysis as a reason for fat loss. Consequently, increasing fatty acid oxidation can be a tentative approach to utilize surplus FFAs. By increasing fatty acid oxidation within adipose tissue, FFAs are oxidized and cannot be re-esterified into TAG [52]. In this present study, there were no significant differences in FFAs levels in the supernatants of TPM-exposed and control 3T3-L1 pre-adipocytes. However, based on the increased glycerol and lipolysis-related mRNA expressions, the current hypothesis is that the produced FFAs from lipolysis are mainly used for fatty acid oxidation and energy production. ACSL1 has a specific function in directing the metabolic partitioning of fatty acids towards β-oxidation [53], and PGC-1α is also involved in mitochondrial fat oxidation [54]. The mRNA levels of ACSL1 and PGC-1α were measured in 3T3-L1 pre-adipocytes and were both up-regulated by TPM in a concentration-dependent manner. The excess of FFAs from enhanced lipolysis are oxidized by mitochondria to produce energy [39], which might contribute to adipocyte senescence and atrophy [55]. The production of ATP was measured, and 25 μg/mL of TPM particularly enhanced the ATP production in the 3T3-L1 pre-adipocytes. At a closer look of the Kyoto Encyclopedia of Genes and Genomes (KEGG) pathway analysis (Figure S9), a significant enrichment of genes related to lipolysis was observed after 25 μg/mL of TPM exposure to the pre-adipocytes. This supports the hypothesis postulated in this present manuscript and the obtained results indicating that TPM exposure to the 3T3-L1 pre-adipocytes enhanced lipolysis, thus, enhancing the fatty acid oxidation, which could be the reason for the fat mass loss and adipose atrophy in the cigarette smoke-exposed mice. This finding was further confirmed by the highly expressed genes involved in mitochondrial oxidative phosphorylation, including complexes I, II, III, and IV, as detected by RNA-sequencing (Figure S10). The postulated mechanism is described in Figure 7. However, it is difficult to adequately extrapolate the present in vitro findings to the in vivo situation as, for example, the duration of cigarette smoke/TPM exposures was not equal.

Importantly, in this current study it was demonstrated for the first time that a relatively short period of chronic cigarette smoke exposure (± 10 weeks) in vivo results in adipose tissue wasting and, to a lesser extent, skeletal muscle wasting. Although whole body lean mass was decreased and skeletal muscle function was deteriorated, no significant changes were observed in skeletal muscle weight and, apart from evidence for reduced protein synthesis signaling, no profound alterations in the expression of markers for muscle protein and mitochondrial turnover were present in the cigarette smoke-induced murine model for COPD. In the in vitro study, enhanced lipolysis and fatty acid oxidation was demonstrated, which might play a role in the fat mass loss and adipose atrophy. More research is needed to elucidate whether fat mass loss and adipose tissue atrophy precedes muscle wasting and to clearly understand the interaction between adipose and muscle tissue. The presented knowledge contributes to a better understanding of the important role of adipose tissue in COPD pathogenesis and progression.

## Data Availability

Data will be made available upon reasonable request.

## Supplementary Materials

The following supporting information can be downloaded at: https://www.mdpi.com/article/10.3390/cells11182893/s1. Figure S1: Timeline of cell differentiation procedure. Figure S2: The total RNA degradation and contamination monitored on 1% agarose gels. Figure S3: Original blots of the protein expression levels of protein turnover markers in soleus muscle via Western blot analysis. Figure S4: Representative pictures of the negative control, isotype control and positive control related to the immunofluorescent staining for CD68 in white adipose tissue. Figure S5: The effect of cigarette smoke exposure on molecular markers of mitochondrial biogenesis and mitochondrial content. Figure S6: The effect of TPM on the viability of 3T3-L1 pre-adipocytes. Figure S7: Optimization of the 3T3-L1 pre-adipocyte differentiation model. Figure S8: Original blots of the protein expression levels of Phospho-HSL, HSL and β-actin in adipocytes after exposing to TPM. Figure S9: Transcriptome sequencing of 3T3-L1 pre-adipocytes exposed to control medium or 25 µg/ml of TPM. Figure S10: KEGG pathway for mitochondrial oxidative phosphorylation of 3T3-L1 pre-adipocytes exposed to control medium or 25 µg/ml of TPM. Table S1. Sequences of the primers
