# Supplementary material for: Effects of Cigarette Smoke on Adipose and Skeletal Muscle Tissue: In Vivo and In Vitro Studies
Source: Cells. 2022 Sep 16;11(18):2893. doi: 10.3390/cells11182893 (PMC9497292; doi:10.3390/cells11182893)
Supplement: Supplementary file 1 [file cells-11-02893-s001.zip › cells-1871204-supplementary.pdf]

cells MDPI

Data supplement

Effects of cigarette smoke on adipose and skeletal muscle tissue: *in vivo* and *in vitro* studies

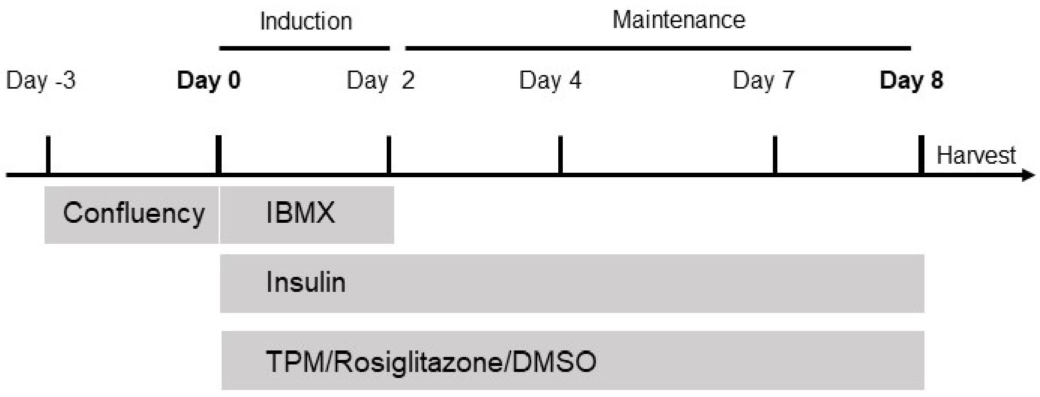

Figure S1. Timeline of cell differentiation procedure. Proliferating preadipocytes were seeded in plates and cultured in phenol red-free medium until reaching confluence (day -3 till day 0). Thereafter, the differentiation medium (insulin with IBMX (day 0 till day 2) and insulin without IBMX (day 2 till day 8). Stimulants (different concentrations of TPM from cigarette smoke, rosiglitazone (positive control), or DMSO (solvent control)) were added from day 0 till day 8 and refreshed on day 2, 4 and 7.

Primers for in vitro		
**Gene name**	Forward Sequence (5′ →3′)	Reverse Sequence (5′ →3′)
**β-actin**	TAA GGC CAA CCG TGA AAA G	ACC AGA GGC ATA CAG GGA CA
**Leptin**	CTG CCC CCC AGT TTG ATG	GCC AGG CTG CCA GAA TTG
**ATGL**	TTA GGA GGA ATG CCC TGC TG	AGC ATG TTG GAA AGG GTG GT
**HSL**	TCC TGG AAC TAA GTG GAC GCA AG	CAG ACA CAC TCC TGC GCA TAG AC
**MGLL**	CGG AAC AAG TCG GAG GTT GAC	CAT TGC TCG CTC CAC TCT TG
**Plin1**	CTG TGT GCA ATG CCT ATG AGA	CTG GAG GGT ATT GAA GAG CCG
**Aqp7**	CTG GAT GAG GCA TTC GTG ACT	TGA TGG CGA AGA GAC ACA GC
**ACSL1**	AAA GAT GGC TGG TTA CAC ACG	CGA TAA TCT TCA AGG TGC CAT T
**PGC-1α**	TGA TGT GAA TGA CTT GGA TAC A	GCT CAT TGT TGT ACT GGT TGG A
**Primers for in vivo**		
**Mstn**	AAC CTT CCC AGG ACC AGG AGA A	TGT CTG TTA CCT TGA CCT CTA AAA ACG G
**Atrogin-1**	GAA GAA ACT CTG CCA GTA CCA CTT C	CCC TTT GTC TGA CAG AAT TAA TCG
**SMART**	AAT TAA TCT GAA AGG CAC TGT GTC	TGA AGA CAG AAT GTC ACA AAC TG
**MuRF-1**	GCG AGG TGG CCC CAT T	GAT GGT CTG CAC ACG GTC ATT
**FoXO-1**	CCT GGA CAT GCT CAG CAG ACA TC	TTG GGT CAG GCG GTT CAT ACC
**REDD1**	CTG ACC CTC GTG CTG CGC CTG	GGA AGC CAG TGC TCA GCG TCA G
**NEDD4**	TCA CTG GCA CAT CTC GGG TG	TCA TAA GGT GGC AAG TCC AGG C
**PGC-1α**	CAA CAA TGA GCC TGC GAA CA	CTT CAT CCA CGG GGA GAC TG
**Tfam**	CCG GCA GAG ACG GTT AAA AA	TCA TCC TTT GCC TCC TGG AA
**Nrf1**	AGC CAC ATT GGC TGA TGC TT	GGT CAT TTC ACC GCC CTG TA
**(Complex II)**	AAT TTG CCA TTT ACC GAT GGG A	AGC ATC CAA CAC CAT AGG TCC
**(Complex III)**	GCA TTC GGA GGG GTT TCC AG	CCG CAT GAA CAT CTC CCC A
**(Complex IV)**	CCA TCC CAG GCC GAC TAA	ATT TCA GAG CAT TGG CCA TAG AA

Table S1. Sequences of the primers

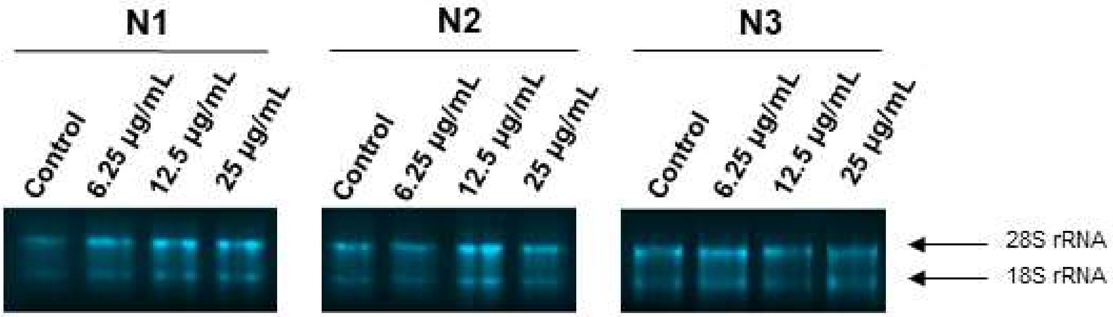

Figure S2. The total RNA degradation and contamination were monitored on 1% agarose gels. Non-denaturing agarose gel (1%) of total RNA from 3T3-L1 pre-adipocytes after exposure to TPM, N=3.

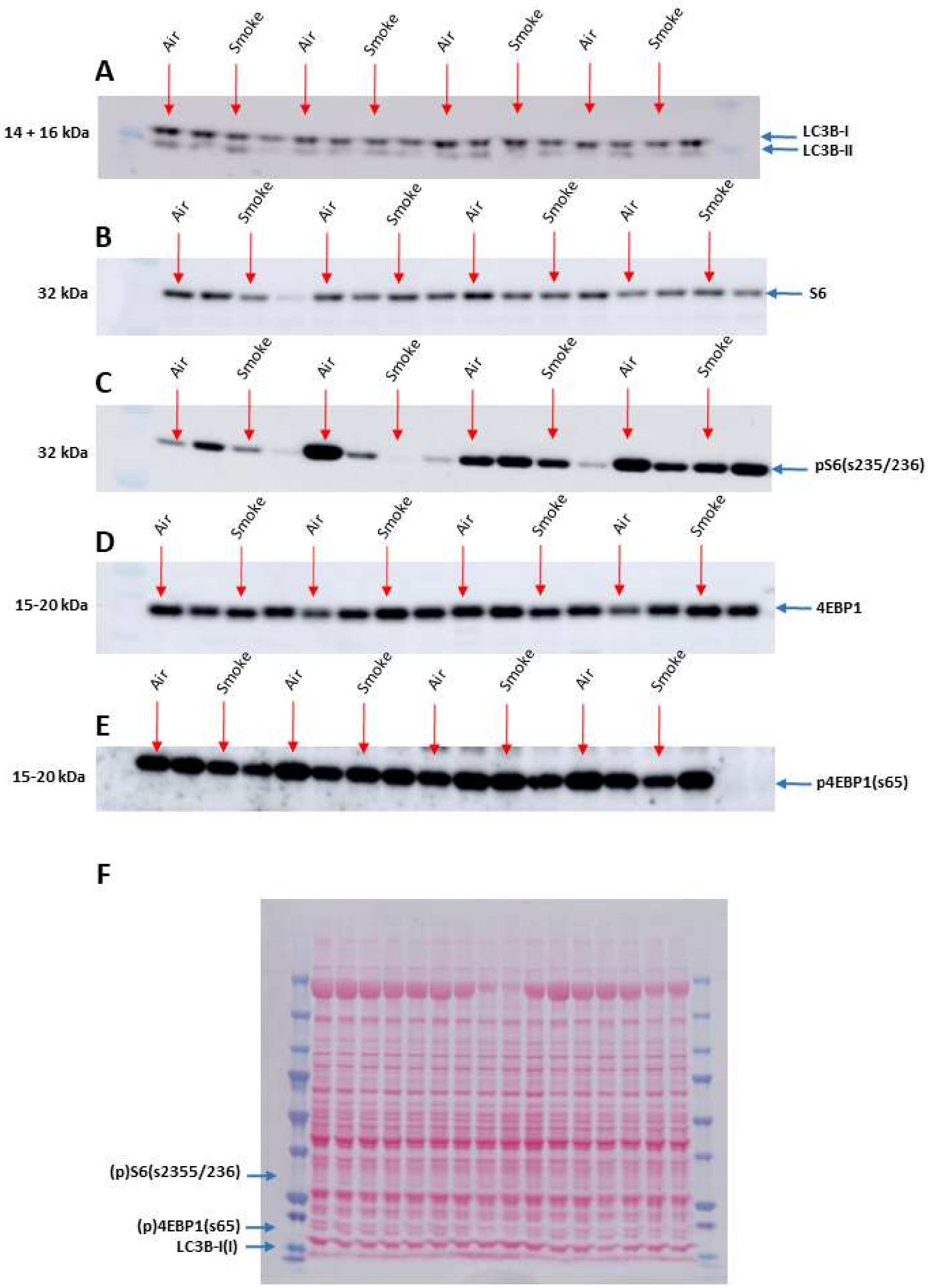

Figure S3: Original blots. Mice were exposed to air or cigarette smoke for 72 days. Protein expression levels of protein turnover markers, LC3B-I(II) (A), S6 (B), pS6(s235/236) (C), 4EBP1 (D and, p4EBP1(s65) (E), were determined in soleus muscle via Western Blot analysis and original blots are depicted, and protein targets were quantified with Ponceau-S (F). N=8 mice/group.

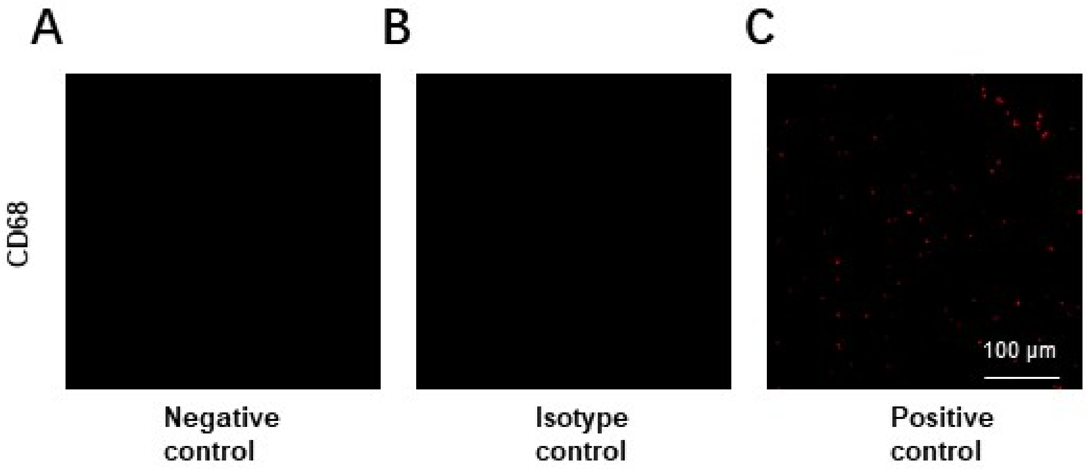

Figure S4. Representative pictures of the negative control, isotype control and positive control related to the immunofluorescent staining for CD68 in white adipose tissue. Mice were exposed to air or cigarette smoke for 72 days. The presence of macrophages in white adipose tissue was investigated by immunofluorescence microscopy using anti-CD68 antibodies, while the negative control by omission of the primary antibodies (A) and rabbit IgG polyclonal isotype control (Abcam, UK) (B) showed no staining and positive control (C) (scale bar = 100 μm).

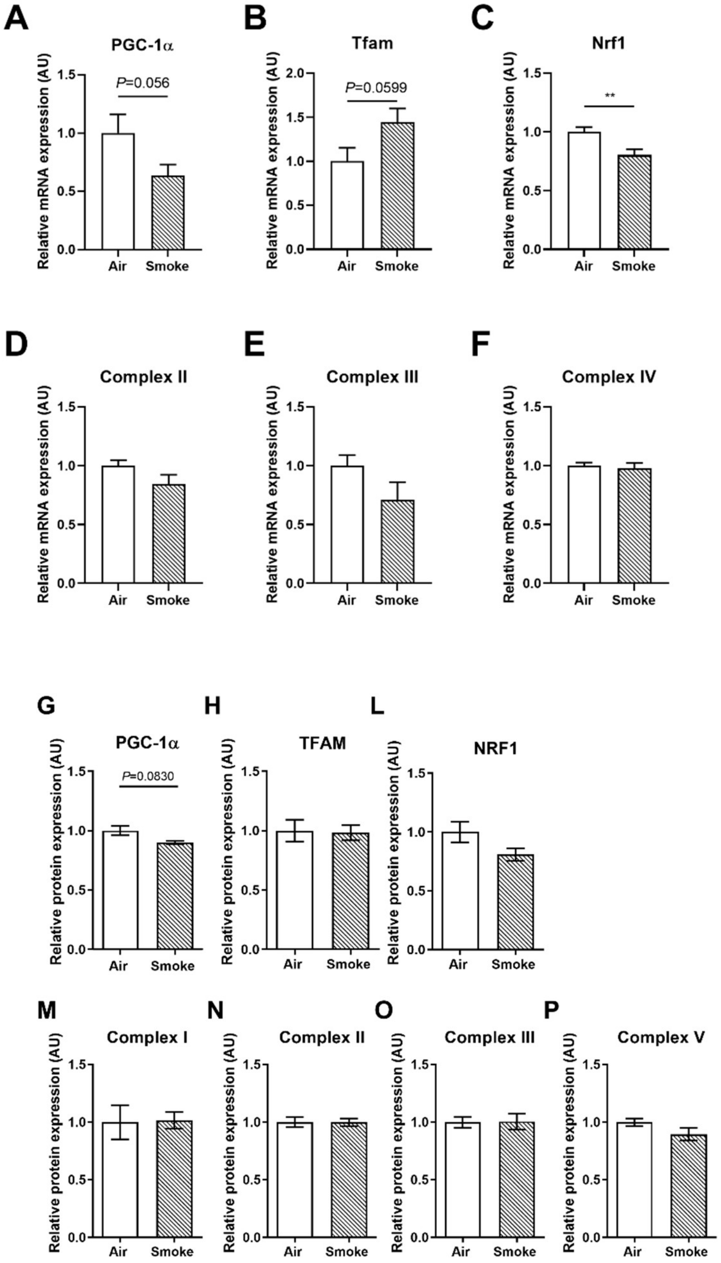

Figure S5. The effect of cigarette smoke exposure on molecular markers of mitochondrial biogenesis and mitochondrial content. Mice were exposed to air or cigarette smoke for 72 days. Gene expression levels of molecular markers for mitochondrial biogenesis PGC-1α (A), Tfam (B), Nrf1 (C), complex II (D), complex III (E) and complex IV (F) were determined in soleus muscle by qRT-PCR. Data was normalized to GeNorm and expressed as fold change compared to control. Protein expression levels of molecular markers for mitochondrial biogenesis PGC-1α (G), TFAM (H), NRF1 (L), complex I (M), complex II (N), complex III (O) and complex IV (P) were determined in soleus muscle by Western blot, and protein targets were quantified with Ponceau-S. Values are represented as mean ± SEM. N=8 mice/group.

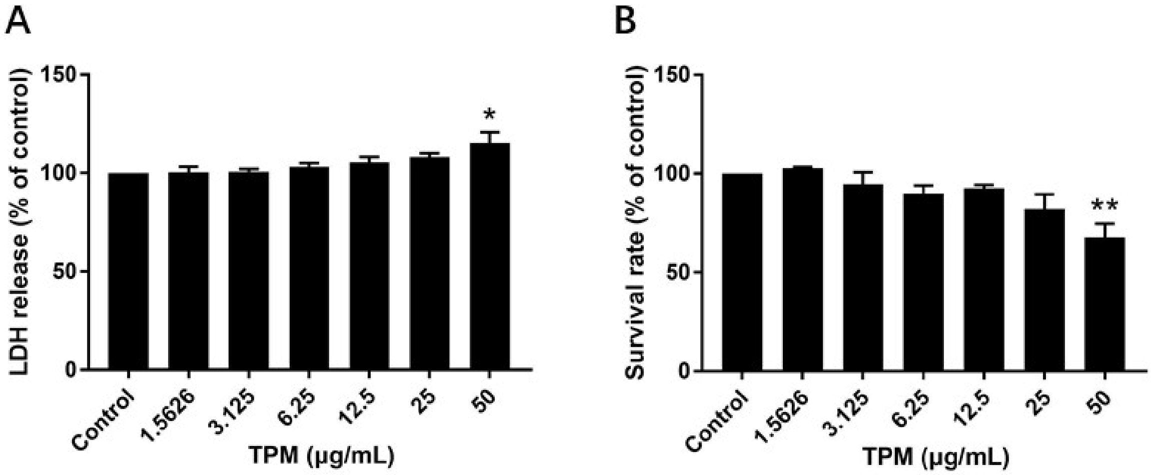

Figure S6. The effect of TPM on the viability of 3T3-L1 pre-adipocytes. The effect of TPM on cell viability of 3T3-L1 pre-adipocytes was evaluated by LDH (A) and MTT (B) assay after exposing 3T3-L1 pre-adipocytes to different concentrations of TPM (1.5626, 3.125, 6.25, 12.5, 25, 50 μg/mL) for 24h. Values are analyzed by one-way ANOVA, Tukey post hoc, and expressed as mean ± SEM, $*p < 0.05$ compared with the control group. N=3.

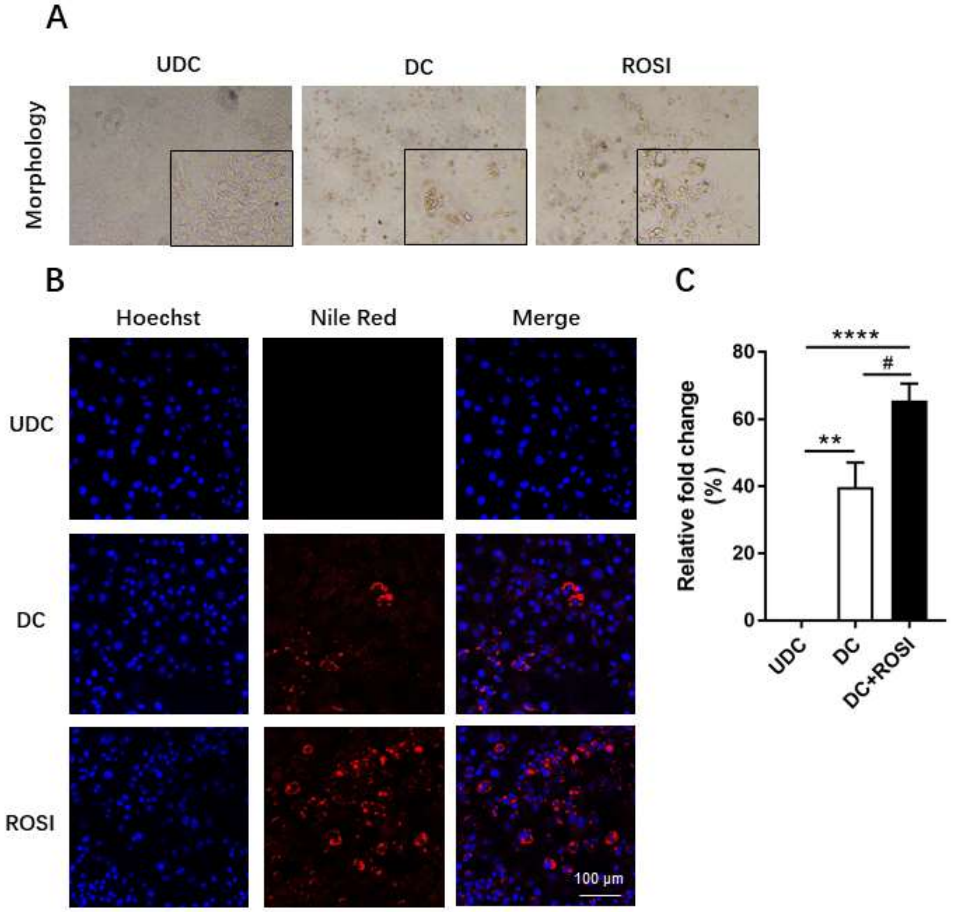

Figure S7. Optimization of the 3T3-L1 pre-adipocyte differentiation model. 3T3-L1 cells, cultured from pre-adipocytes were differentiated with insulin and IBMX to achieve the mature adipocyte phenotype following an 8-day differentiation protocol (2 days induction of differentiation and 5 days maintenance of differentiation). Rosiglitazone (ROSI) was added as the positive control. The cell morphology was imaged on day 8 (A). In the undifferentiated control (UDC) group, the cells displayed a fibroblast phenotype, whereas the cells in the differentiated control (DC) group were more rounded and compact. These rounded and compact cells were even more obvious in the positive control group incubated with rosiglitazone. Nuclei and lipid droplets were stained with a Hoechst (blue color) and Nile red (red color) dye, respectively (B). The Nile red fluorescent intensity was read and quantified via a fluorescent reader (C). The expression of the lipid droplets was around 40% and 60% higher in the DC group and positive control group, respectively as compared to the UDC group. Values are analyzed by one-way ANOVA, Tukey post hoc and expressed as mean ± SEM, N = 4. **$p < 0.01$, ****$p < 0.001$, compared with UDC; #$p < 0.05$, compared with DC.

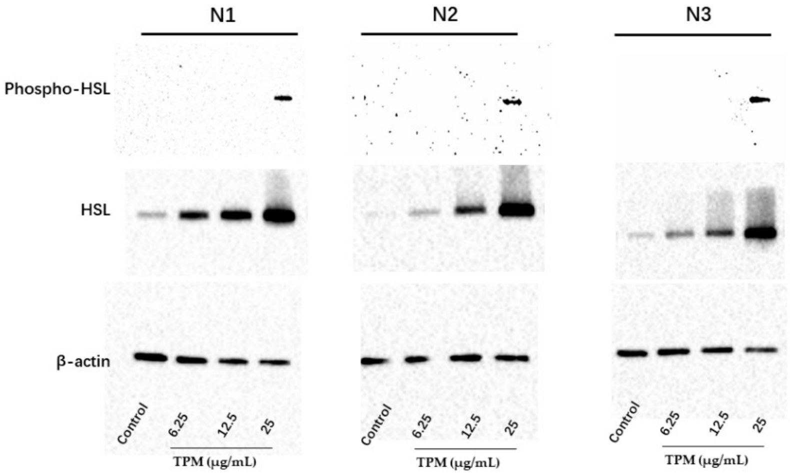

Figure S8. Original blots of adipocytes N=3 replicates. Protein expression levels of Phospho-HSL, HSL, and β-actin were determined in adipocytes after exposure to TPM.

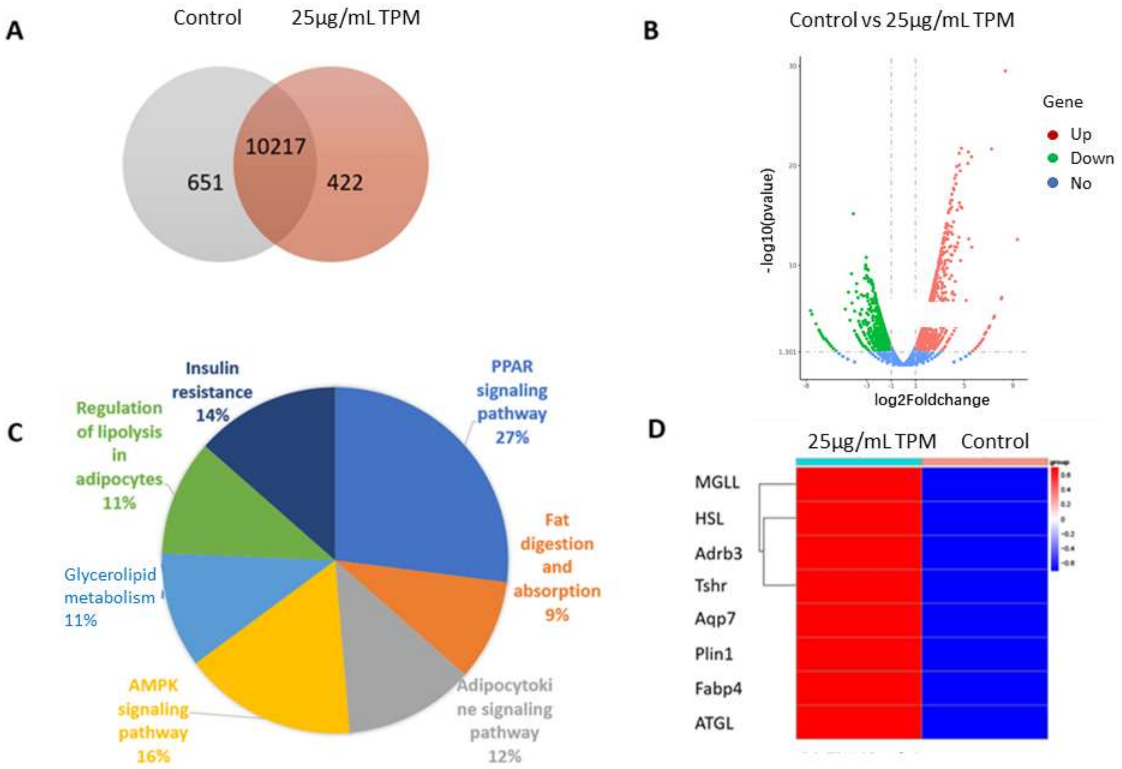

Figure S9. Transcriptome sequencing of 3T3-L1 pre-adipocytes exposed to control medium or 25 μg/ml TPM. Control medium and 25 μg/ml TPM were added to the 3T3-L1 pre-adipocytes during the differentiation procedure of 8 days. RNA was isolated and transcriptome sequencing was performed. Venn chart of genes with significantly altered mRNA expression (A) | log2 FoldChange |>2; Volcanic chart of genes with significantly altered mRNA expression (B)| log2 FoldChange |>2; KEGG pathway enrichment analysis of genes with significantly altered mRNA expression (C)| log2 FoldChange |>5; Heatmap of genes involved in lipolysis (D) (N=1). Adrb3: Adrenoceptor beta 3; Tshr: Thyroid stimulating hormone receptor; Fabp4: Fatty Acid Binding Protein 4.

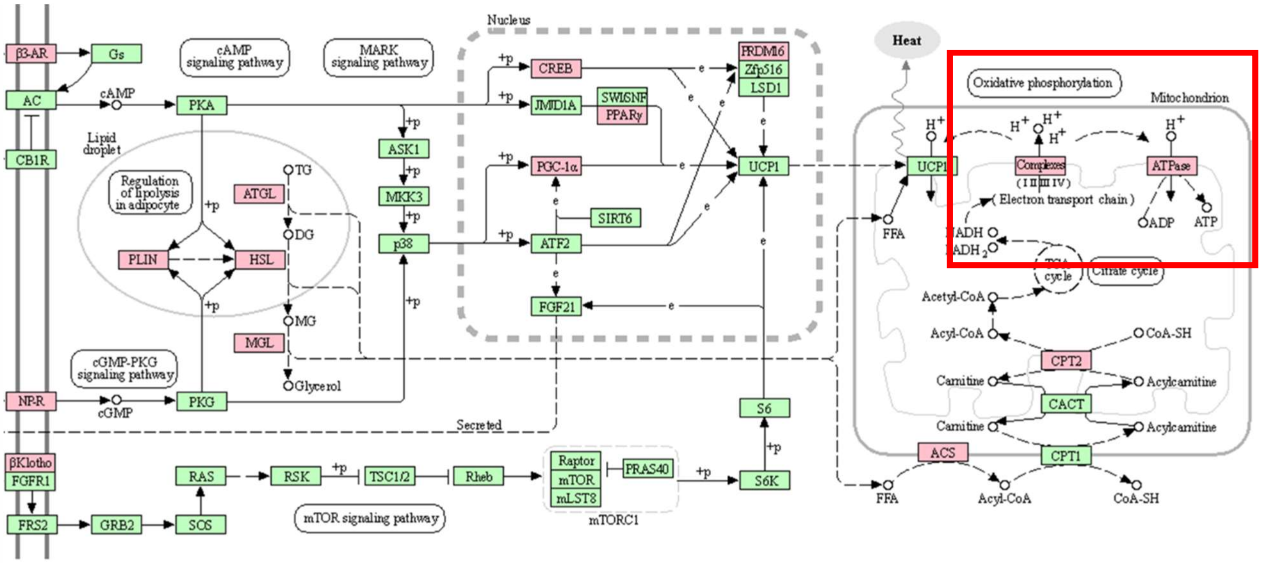

Figure S10. KEGG pathway for mitochondrial oxidative phosphorylation of 3T3-L1 pre-adipocytes exposed to control medium or 25 μg/ml TPM. Control medium and 25 μg/ml TPM were added to the 3T3-L1 pre-adipocytes during the differentiation procedure of 8 days. RNA was isolated and transcriptome sequencing was performed. KEGG pathway enrichment analysis of genes involved in mitochondrial oxidative phosphorylation (Genes labeled in pink were significantly altered, Fold change >2).
